# Optimised production of an anti-fungal antibody in *Solanaceae* hairy roots to develop new formulations against *Candida albicans*

**DOI:** 10.1186/s12896-020-00607-0

**Published:** 2020-03-12

**Authors:** Marcello Catellani, Chiara Lico, Mauro Cerasi, Silvia Massa, Carla Bromuro, Antonella Torosantucci, Eugenio Benvenuto, Cristina Capodicasa

**Affiliations:** 1 Department of Sustainability, Laboratory Biotechnologies, ENEA, Casaccia Research Center, Via Anguillarese 301, 00123 Rome, Italy; 2 Department of Infectious, Parasitic and Immune-Mediated Diseases, Istituto Superiore di Sanità, Rome, Italy

**Keywords:** Fungal infections, Hairy roots, Molecular farming, Proteolytic activity, Recombinant antibody, Rhizosecretion

## Abstract

**Background:**

Infections caused by fungi are often refractory to conventional therapies and urgently require the development of novel options, such as immunotherapy. To produce therapeutic antibodies, a plant-based expression platform is an attractive biotechnological strategy compared to mammalian cell cultures. In addition to whole plants, hairy roots (HR) cultures can be used, representing an expression system easy to build up, with indefinite growth while handled under containment conditions.

**Results:**

In this study the production in HR of a recombinant antibody, proved to be a good candidate for human immunotherapy against fungal infections, is reported. Expression and secretion of this antibody, in an engineered single chain (scFvFc) format, by HR from *Nicotiana benthamiana* and *Solanum lycopersicum* have been evaluated with the aim of directly using the deriving extract or culture medium against pathogenic fungi. Although both *Solanaceae* HR showed good expression levels (up to 68 mg/kg), an optimization of rhizosecretion was only obtained for *N. benthamiana* HR. A preliminary assessment to explain this result highlighted the fact that not only the presence of proteases, but also the chemical characteristics of the growth medium, can influence antibody yield, with implications on recombinant protein production in HR. Finally, the antifungal activity of scFvFc 2G8 antibody produced in *N. benthamiana* HR was evaluated in *Candida albicans* growth inhibition assays, evidencing encouraging results.

**Conclusions:**

Production of this anti-fungal antibody in HR of *N. benthamiana* and *S. lycopersicum* elucidated factors affecting pharming in this system and allowed to obtain promising ready-to-use immunotherapeutics against *C. albicans*.

## Background

Besides a source of nutrition, plants have always been an inexhaustible resource of useful materials and molecules. A further exploitation comes from biotechnology that gives the opportunity to express recombinant, high-value proteins in plants. “Plant pharming” offers the possibility to produce biologics, and, among these, complex proteins such as immunoglobulins, in an economical, easy and safe manner. Since the first report of antibody production in tobacco [1], in the last two decades several antibodies, intended for anti-cancer or anti-infective therapeutic applications, have been expressed using different expression systems and various host plants [2, 3]. Recently, a plant-made anti-HIV human IgG produced in *Nicotiana tabacum*, in a cGMP compliant process, has successfully completed a Phase 1 safety trial as a microbicide formulation delivered to vaginal mucosa [4]. Moreover, during the 2014 Ebola outbreak in Liberia, an experimental cocktail of three antibodies, named ZMapp, produced in plants [5] according to the cGMP and using RAMP platform (Rapid Antibody Manufacturing Platform), was made available on an emergency use basis for compassionate treatment of a patient population of seven individuals, five of which recovered [6]. Hence, this and other examples showcase the approach of producing biologicals in plants as a valuable low-cost alternative to synthesize pharmaceuticals to combat pathogenic agents.

The most widely used systems to produce such biologics utilize plant biomass and transient expression techniques based on viral infections or infiltration with *Agrobacterium tumefaciens* that allows the best yields of recombinant proteins [7, 8]. However, plant tissue cultures, either suspension cell culture or organ cultures (e.g. hairy roots), can also be exploited for biopharmaceutical proteins production, offering several advantages as easy containment and scale-up in industrial bioreactors [9]. In particular, HR are obtained by infecting plant tissues with *Rhizobium rhizogenes* (formerly *Agrobacterium rhizogenes*), a plant pathogen that transfers to plant cells a set of genes inducing the formation of roots capable of indefinite growth. HR have a fast multiplication rate, are genetically stable and relatively simple to maintain in hormone-free media. In addition, recombinant proteins can be designed to be secreted into the media, facilitating recovery and lowering the cost of their purification phases, required for valuable therapeutic proteins [10]. Rhizosecretion of foreign proteins has been already investigated for reporter proteins such as GFP [11], but also for biopharmaceuticals, as LTB [12] or monoclonal antibodies [13].

We recently demonstrated that chimeric murine-human antibodies directed against the fungal cell wall polysaccharide β1–3 glucan (in form of full IgG or scFvFc) are able to confer a significant protection in vivo against systemic and mucosal (vaginal) infections caused by the fungal pathogen *C. albicans* [14]. These infections are a growing health concern, especially for immunocompromised subjects, often refractory to conventional therapies, and would urgently require the development of novel therapeutic options. To this end, the use of chimeric anti-beta-glucan antibodies as a replacement or an integration of classical drug therapy could represent a new and promising immunotherapeutic approach. To reduce production costs of conventional mammalian cell-based systems, often expensive and not easily scalable, the chimeric anti-fungal IgG and scFvFc 2G8 were transiently expressed in *N. benthamiana* plants, a well-consolidated platform to produce biopharmaceuticals [15].

In this work, we explored HR derived from *N. benthamiana* and *S. lycopersicum* plants, as alternative platforms to produce the antifungal chimeric scFvFc 2G8. Antibody production in HR allows steady process controls to regulate growth and product formation, besides simplifying purification steps, compared to whole plant transient expression approaches. We evaluated the scFvFc 2G8 expression level in whole root tissues or secreted in culture medium of both *N. benthamiana* and *S. lycopersicum* HR, highlighting and giving a clue about the differences in antibody production by these two *Solanaceae* species.

Moreover, we verified that antibodies purified from HR retained the same specific antigen binding ability of scFvFc 2G8 transiently produced in leaves. Finally, we tested both crude extract and concentrated culture medium of 2G8-producing *N. benthamiana* HR for inhibitory activity against the growth of the fungal pathogen *C. albicans,* with the aim to devise a possible ready-to-use antimicrobial immunotherapeutic for topical application.

## Results

### Generation of *N. benthamiana* and *S. lycopersicum* HR producing 2G8 antibody

**Fig. 1:**
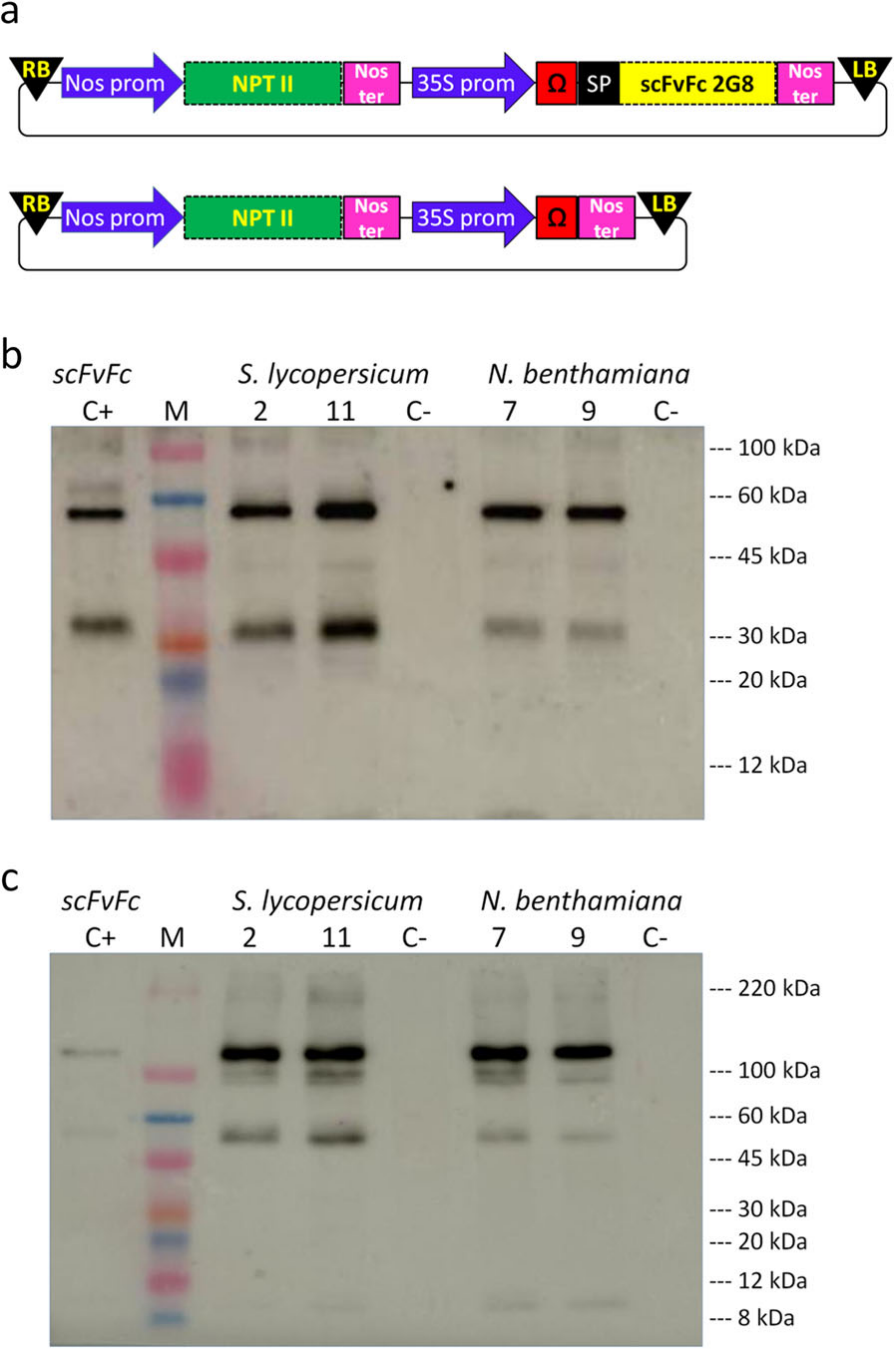
Generation of *N. benthamiana* and *S. lycopersicum* HR expressing scFvFc 2G8. **a** Schematic representation of construct containing the scFv format of the mAb 2G8 fused to the Fc-encoding sequence of the human IgG1 chain or vector used for irrelevant control (pBIc-). SP: signal peptide sequence derived from an embryonic immunoglobulin to direct the recombinant antibody to the secretory pathway [16]; Ω: TMV translational enhancer [17]; 35S prom: Cauliflower Mosaic Virus 35S promoter; Nos ter: Nopaline synthetase terminator sequence. Western blot analysis of TSP (5 μg) extracted from HR expressing scFvFc 2G8 or C- separated on a 10% Tris/glycine gel by SDS-PAGE in reducing (**b**) or non-reducing (**c**) conditions. C+: 25 ng of purified scFvFc 2G8. Blots were detected with anti-human IgG1-antibody conjugated to HRP

To generate HR producing the recombinant anti-β-glucan scFvFc 2G8, *N. benthamiana* and *S. lycopersicum* leaf explants were infected with *R. rhizogenes* harbouring the pBIM2G8 vector containing the scFvFc 2G8 gene (Fig. 1a). Twenty-six and 23 transformed HR clones were obtained for *N. benthamiana* and *S. lycopersicum,* respectively, and among these 3 clones for *N. benthamiana* and 10 for *S. lycopersicum* were found to express the scFvFc 2G8 at higher levels (over 10 ng per mg of root), as determined by a functional semi-quantitative ELISA using β1,3-glucan (laminarin) as the target antigen (data not shown). The antibody yields for the two top producer clones for each species (#7 and #9 for *N. benthamiana* and #2 and #11 for *S. lycopersicum*) are reported in Table 1. The scFvFc yield, measured at day 14 of the culture cycle, was similar for clones #7, #9 of *N. benthamiana* and #2 of *S. lycopersicum*, (approximately 23, 18 and 25 μg/g root fresh weight (FW), respectively, and above 1% of extracted total soluble proteins, TSP), but much higher for the *S. lycopersicum #*11 clone (68 μg/g root FW and 2.4% of extracted TSP).

**Table 1:** Crude yield of scFvFc 2G8 produced HR. Concentration of functional antibody produced in the extracts of roots, sampled at day 14 of culture cycle, was calculated by quantitative ELISA and values indicate average yield ± standard error (SE, n = 3). The yields are reported as Ab μg for g of fresh weight (FW) or Ab percentage in total soluble proteins (TSP) extracts of HR.

	scFvFc 2G8 Yield	
	μg/g	% TSP
***N. benthamiana***		
Clone 7	**23.12 ± 0.59**	**1.25**
Clone 9	**18.35 ± 1.62**	**1.20**
***S. lycopersicum***		
Clone 2	**25.17 ± 0.60**	**1.36**
Clone 11	**68.22 ± 3.57**	**2.44**

The integrity and correct assembly of the scFvFc 2G8 expressed by these clones was verified by Western blot analysis of TSP in root extracts (Fig. 1). All clones exhibited a comparable band pattern, which overlapped that shown by the purified scFvFc loaded on the same gel. In reducing conditions (Fig. 1b), we detected a clear and preponderant band at the expected molecular weight (MW ~ 55 kDa) for the reduced scFvFc, and two minor additional bands of lower MW, the major of which (MW 30 kDa) likely corresponding to the Fc portion of the recombinant antibody. Analysis in non-reducing conditions (Fig. 1c) showed a prevailing band at the expected molecular mass (~ 110 kDa) for the assembled scFvFc 2G8 and three additional bands of lower molecular weight, less intense, probably corresponding to antibody degradation fragments.

### Characterization of scFvFc 2G8 expressed in HR

The scFvFc 2G8 produced in HR of *N. benthamiana* and *S. lycopersicum* were purified and their functionality was tested. Antibody purification was performed from a clarified root extract by affinity chromatography on protein A and a dialysis/concentration step by ultrafiltration, with a final recovery of 75% of antibody.

**Fig. 2:**
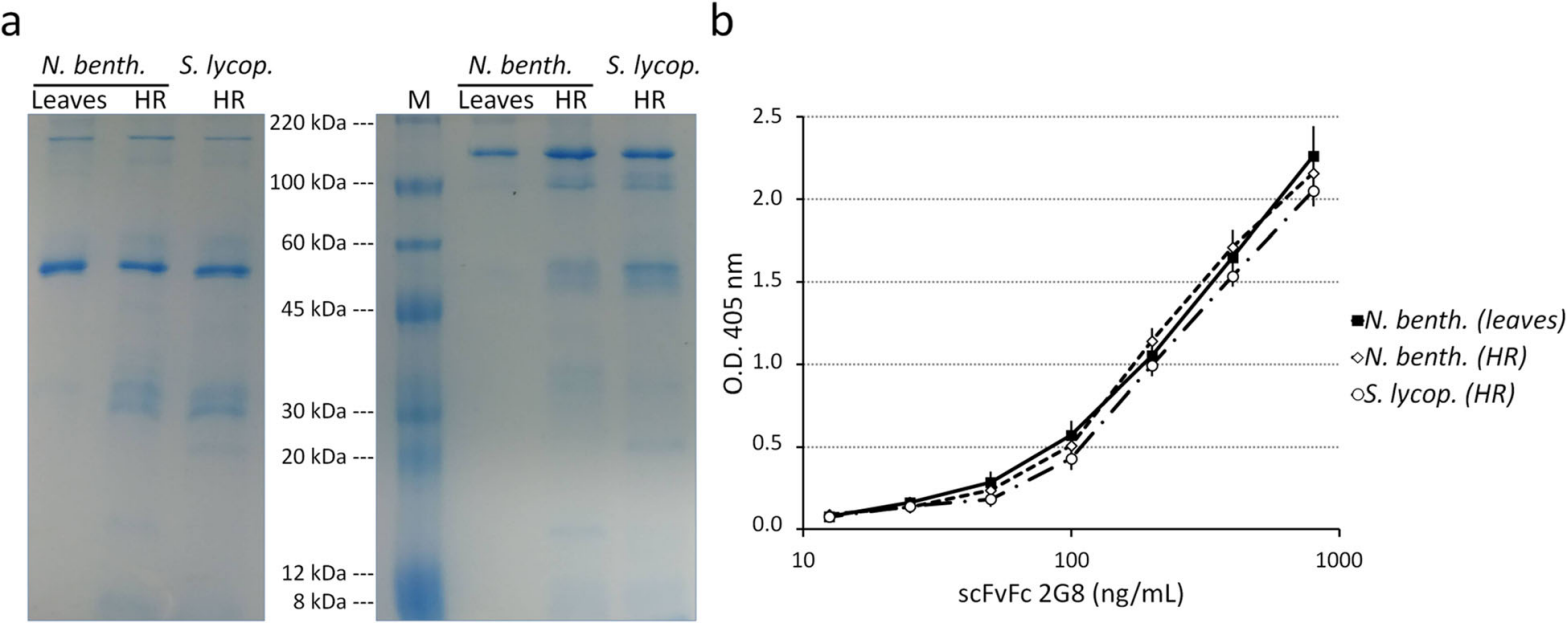
Comparison of scFvFc 2G8 purified from HR and *N. benthamiana* leaves. **a** SDS-PAGE of antibodies purified by protein A-based affinity chromatography from extracts of *N. benthamiana* and *S. lycopersicum* HR and agro-infiltrated *N. benthamiana* leaves. The purified antibodies (400 ng) were loaded on 10% SDS polyacrylamide gel under reducing (left part of the gel) and non-reducing conditions (right part of the gel) and stained with colloidal Coomassie blue. M: ColorBurst Electrophoresis Marker (M.W. 8000–220,000 Da)(Sigma-Aldrich). **b** Functional binding of scFvFc 2G8 recombinant antibodies purified from HR and *N. benthamiana* leaves to immobilized laminarin assessed by ELISA. Anti-human γ chain antibody conjugated to alkaline phosphatase was used as secondary reagent for assay development. Specific binding (O.D. 405 nm) is plotted as function of the scFvFc 2G8 concentration (ng/mL) after subtraction of negative controls absorbance. Values represent average ± standard deviation (*n* = 3)

Both purified antibodies, resolved by SDS-PAGE (Fig. 2a), showed an almost identical pattern with a preponderant band at about 55 or 110 kDa, under reducing and non-reducing conditions respectively, corresponding to a single chain or assembled scFvFc 2G8. Additional faint bands of lower molecular weight are present, more evident and numerous in *S. lycopersicum*, indicating the occurrence of a slight antibody degradation. Densitometric analysis of the Coomassie blue-stained gels showed that the intact antibody (band at 110 kDa) derived from HR of *N. benthamiana* and *S. lycopersicum* represented about 90 and 79%, respectively, of total purified proteins.

To assay the antigen recognition, we compared the binding profiles of the antibodies purified from HR and from *N. benthamiana* leaves to the β-glucan antigen laminarin (an algal standard β-glucan mimetic of fungal β-glucan) by ELISA (Fig. 2b). As expected, all antibodies reacted with laminarin in an almost identical dose-dependent manner. The scFvFc produced in leaves showed a little higher intensity of binding (statistically not significant) probably due to the slight degradation of both HR-purified antibodies.

### Optimisation of antibody rhizosecretion

**Fig. 3:**
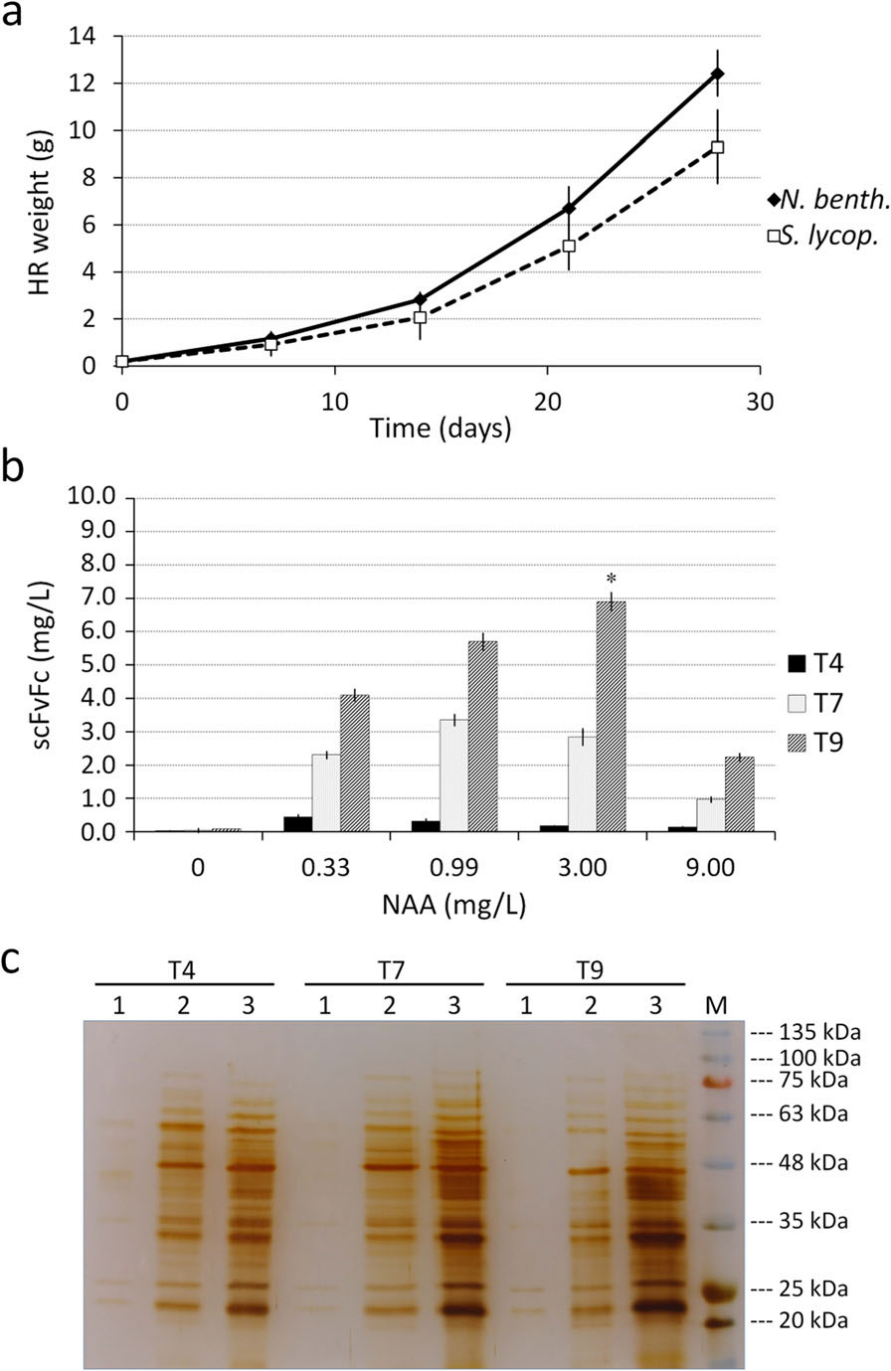
Optimisation of culture medium for *N. benthamiana* HR. **a** Growth curves of *N. benthamiana* (clone 7) and *S. lycopersicum* (clone 11) HR liquid cultures expressing scFvFc 2G8. Growth was followed for 28 days after HR inoculum (200 mg). **b** Quantification by ELISA of scFvFc 2G8 secreted by *N. benthamiana* #7 in MS culture medium supplemented with KNO_3_ 10 g/L, and various concentrations of NAA. Different time intervals (T: 4, 7 and 9 days) from NAA administration have been considered. Values represent average ± SE (n = 3). Statistically significant difference (*P* < 0.05, Student’s t test) at 3 mg/L of NAA at T9 with respect to the other concentrations is marked by an asterisk. **c** Comparison by silver stained SDS-PAGE of proteins rhizosecreted by *N. benthamiana* HR C- at different time intervals from growth regulators addition. For each sample, a volume of culture medium normalized with root biomass (25 mg) was loaded. Lane 1: MS without growth regulators; lane 2: optimized MS supplemented with KNO_3_ 10 g/L, NAA 3 mg/L; lane 3: MS supplemented with KNO_3_ 12 g/L, NAA 19 mg/L, and PVP 1.5%. M: Prestained Protein Marker Sharpmass VI (EuroClone)

The biomass production and growth kinetic of two best producing clones of each plant species were evaluated (Fig. 3a). Growth curves of clone #7 and clone #11 obtained along a 28 days culture cycle showed that tomato HR grew slightly slower than *N. benthamiana* clone, resulting in a lower biomass yield at the end of culture cycle.

Having fused the scFvFc 2G8 to a secretory signal peptide (a sequence deriving from an embryonic mouse immunoglobulin [14, 16]), rhizosecretion of the antifungal Ab in culture medium of the best expressing clones obtained from *N. benthamiana* (#7) and *S. lycopersicum* (#11) was verified. In line with previous works, HR grown in standard liquid medium (Murashige and Skoog, MS) secreted only very low (*N. benthamiana*) or undetectable (*S. lycopersicum*) levels of the antibody (data not shown). Therefore different medium formulations, enriched with nitrate (KNO_3_) and the growth regulator 1-naphtaleneacetic acid (NAA), were tested to improve antibody accumulation. Initially, we used the medium and method developed by Hakkinen and colleagues [13] to optimize the secretion of a full antibody in *N. tabacum* HR (MS medium supplemented with KNO_3_ 12 g/L, NAA 19 mg/L and polyvinylpyrrolidone, PVP, 1.5%). However, roots cultured in this medium, in particular HR from *S. lycopersicum*, showed unsatisfactory growth rates and already at the end of the first week from subculture, tissues developed an unhealthy general aspect with a brownish phenotype attributable to premature aging and necrosis (data not shown).

To define the lower amount of KNO_3_ and NAA required to obtain a good Ab secretion while preserving HR fitness, the composition of the rhizosecretion medium was changed, adding one element at a time. Different concentration of KNO_3_ (5 and 10 g/L) were firstly tested: a slight increase in the amount of secreted antibody, corresponding to a final yield of 40 and 70 μg/L, was obtained in the culture medium added with 5 and 10 g/L of KNO_3_, respectively, after 9 days, but only for *N. benthamiana*, while *S. lycopersicum* HR did not benefit at all from this change in medium composition. Fixing the KNO_3_ concentration at 10 g/L, the medium was then supplemented with four different concentrations of NAA (from 0.33 to 9 mg/L), each tested in duplicate, analysing the amount of secreted scFvFc 2G8 at 4, 7 and 9 days of root cultivation. For *N. benthamiana* HR, a significant increase in antibody accumulation, at all NAA concentrations, was observed over time, reaching the maximum level at day 9 (Fig. 3b). The peak of antibody accumulation occurred in the medium supplemented with NAA 3 mg/L, resulting in a yield of ~ 7 mg/L, while a severe decrease was observed at 9 mg/L NAA (Fig. 3b). Here again, all tested samples of *S. lycopersicum* resulted negative for antibody rhizosecretion. Moreover, in our optimised medium both *Solanaceae* HR, despite having a slightly slower growth, showed a general healthy phenotype, comparable to HR grown in standard MS.

We also compared the pattern of secreted proteins over time upon HR growth in MS without growth regulators, in the herein optimised MS supplemented with KNO_3_ 10 g/L and NAA 3 mg/L and in the MS medium supplemented with KNO_3_ 12 g/L, NAA 19 mg/L and PVP 1.5%, initially used (Fig. 3c). The SDS-PAGE analysis of the protein content of different media revealed that in standard MS very few proteins are present in culture medium, while in both MS media supplemented with growth factors a greater number of secreted proteins was found. Nonetheless, in our optimised MS, the total amount of proteins was lower and did not increase over time (Fig. 3c).

### Influence of pH and peptidases on antibody accumulation in the HR growth medium

**Fig. 4:**
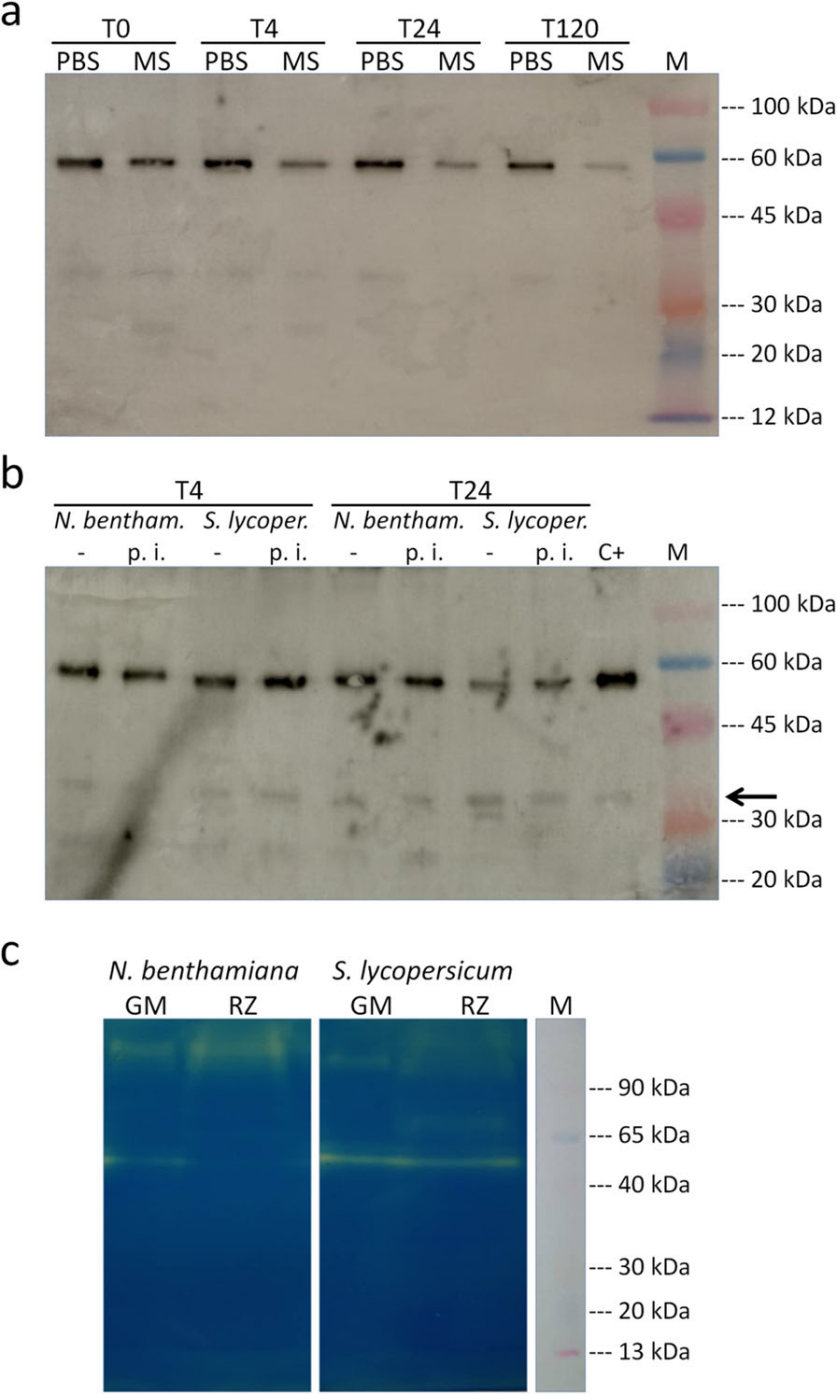
Evaluation of scFvFc 2G8 stability and proteolytic activity in *N. benthamiana* or *S. lycopersicum* HR culture media. Western blot of purified scFvFc 2G8 (150 ng/lane) incubated for different times (0, 4, 24 and 120 h) in MS medium or in PBS (**a**) or incubated for 4 or 24 h in medium where HR C- of the indicated species were grown (**b**), with or without the addition of peptidase inhibitors (p.i.), resolved by 10% Tris-Glycine gel in reducing conditions. C+: 150 ng of scFvFc 2G8. Blots were detected with goat anti-human IgG (γ-chain specific) HRP-conjugated antibody. The partial scFvFc 2G8 degradation, mediated by *S. lycopersicum* proteases, was indicated with solid arrow. **c** Zymogram of *N. benthamiana* and *S. lycopersicum* growth media (GM) concentrated 4 fold and rhizosecretome (RZ) separated on a 10% SDS polyacrylamide gel supplemented with gelatin (0.05%) and stained with colloidal Coomassie blue. M: ColorBurst Electrophoresis Marker (M.W. 8000–220,000 Da)(Sigma-Aldrich)

To elucidate possible causes of the lowest amount of scFvFc 2G8 accumulated in the growth medium compared to that found inside the roots, particularly seen in *S. lycopersicum* HR, antibody stability in the HR growth medium was investigated. Firstly, we evaluated the effect of the slightly acidic pH (5.8) of the MS-based growth medium. Thus, the purified antibody was diluted in MS medium or in PBS pH 7.2, incubated at room temperature for 4, 24 and 120 h and then analysed by SDS-PAGE in reducing conditions and Western blot (Fig. 4a). In the sample with the scFvFc 2G8 diluted in MS medium, a clear reduction over time of the intensity of the band corresponding to the antibody (~ 55 kDa), most evident at 120 h, was observed. This reduction in intensity did not correspond to an increase in number or intensity of the bands of lower MW, suggesting that it was likely due to precipitation of the antibody rather than to its degradation. Since one major limitation of secretion-based systems is the occurrence of proteolytic events affecting the products, we checked for possible antibody degradation mediated by peptidases released by HR during growth. To this aim, a purified scFvFc 2G8 was incubated for 4 or 24 h in the medium in which *N. benthamiana* or *S. lycopersicum* HR transformed with the empty vector (pBIc-, hereinafter HR C-) were grown. As a specificity control, duplicate sample were supplemented with the proteases inhibitors PMSF (specific for serine proteases) and EDTA (specific for metalloproteases). All the samples were then analysed by SDS-PAGE in reducing conditions and Western blot. As shown in Fig. 4b, only a slight antibody degradation occurred when the scFvFc was incubated for 4 h in either *N. benthamiana* or *S. lycopersicum* growth medium. Differently, after 24 h of incubation, an appreciable degradation (partially inhibited by PMSF and EDTA) was observed in the scFvFc incubated in *S. lycopersicum*, but not in *N. benthamiana* growth medium. To get a first insight into peptidases secreted by *N. benthamiana* and *S. lycopersicum* HR, the growth medium and the rhizosecretome were analysed by zymography with gelatin as substrate [18]. As shown in Fig. 4c, the peptidases activity seems different between *N. benthamiana* and *S. lycopersicum* samples, and a more evident gelatin degradation appeared only for the latter HR species in both concentrated growth medium and rhizosecretome, showing different band patterns.

On the whole, these experiments suggest that antibody produced in roots, when secreted, in part precipitates due to the low pH of the growth medium, and in part it is degraded by the peptidases present in the secretome (especially in *S. lycopersicum*).

### *Candida albicans* growth inhibition by HR-produced scFvFc 2G8 antibody

**Fig. 5:**
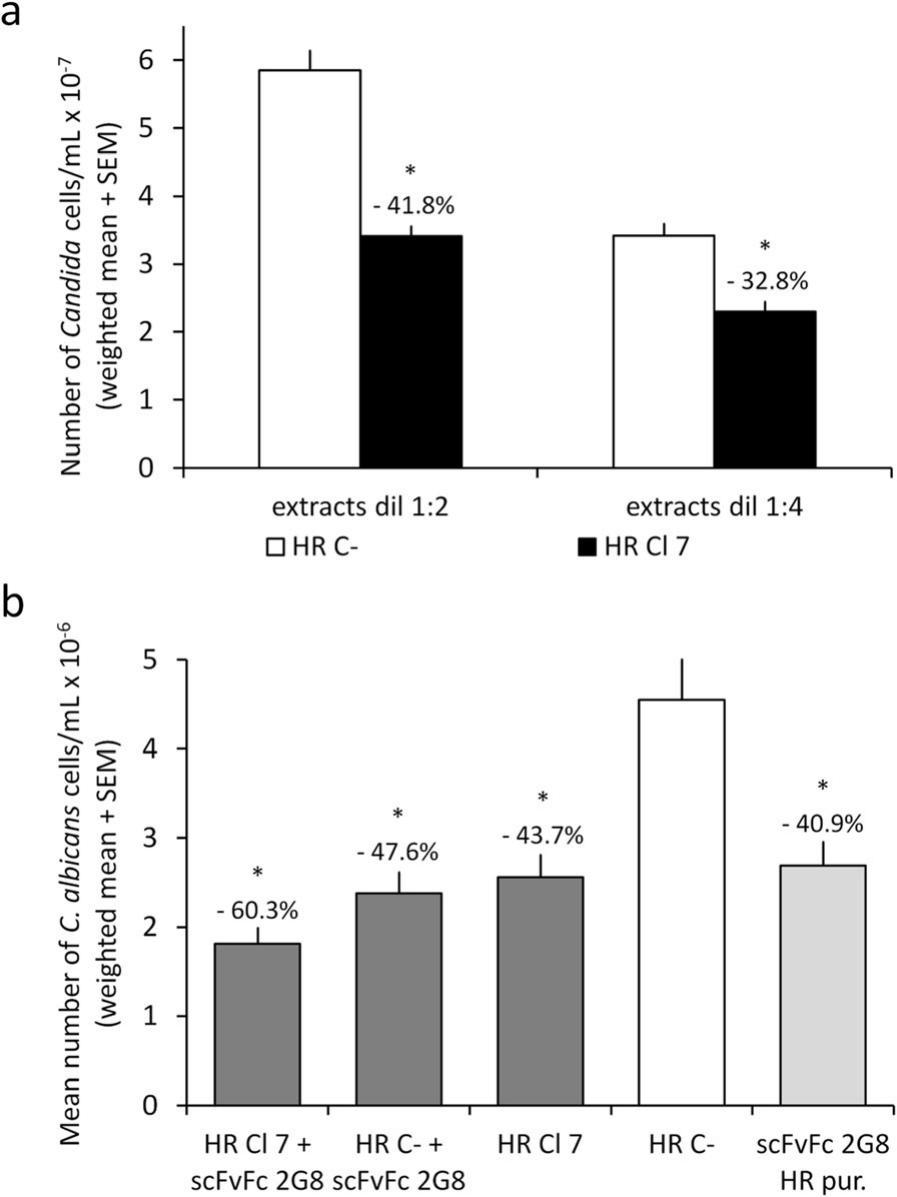
Inhibition of *C. albicans* growth by extracts or culture medium of *N. benthamiana* scFvFc 2G8 HR. *Candida albicans* cells were cultured at 37 °C in the presence of the indicated HR concentrated extracts. **a***Candida albicans* cells (10^3^/mL) were cultured at 37 °C in the presence of concentrated extracts of *N. benthamiana* HR expressing (HR Cl 7) or not expressing (HR C-, negative control) the 2G8 antibody. **b** The same number of fungal cells were cultured in presence of a concentrated culture medium of *N. benthamiana* HR expressing the 2G8 antibody (HR Cl 7, final antibody concentration 150 μg/mL) or with an identically concentrated culture medium of *N. benthamiana* HR C- (negative control) or with a scFvFc 2G8 purified from *N. benthamiana* HR (scFvFc 2G8 HR pur, final antibody concentration 200 μg/mL). When indicated (+ scFvFc 2G8), the scFvFc 2G8 purified from *N. benthamiana* leaves (final concentration 200 μg/mL) was added to HR concentrated culture media. All conditions were assayed in triplicate. After 24 h of incubation, fungal growth was estimated by enumerating fungal cells with a standard CFU counts. Values in the graphs are mean numbers of fungal cells found in the different cultures at the end of the incubation period. Percent growth inhibition values were calculated by comparison to negative controls (cultures growth in the presence of HR C- preparations) and are reported at the end of the relevant bar. Statistically significant differences (P < 0.05, two-tailed Student’s t test) with respect to negative controls are marked by asterisks

Finally, we verified whether the concentrated protein extracts and growth medium of scFvFc 2G8-expressing *N. benthamiana* HR or the purified *N. benthamiana* HR-derived scFvFc 2G8 could directly inhibit the growth of the fungal pathogen *C. albicans*, as previously reported for the antibody obtained transiently in *N. benthamiana* leaves [14]. For these experiments, an identical number of fungal cells (10^3^/mL) was inoculated in the presence of extracts or culture media, concentrated 15 and 25 times respectively, from HR expressing scFvFc 2G8 and HR C-, or with scFvFc 2G8 purified from *N. benthamiana* HR extracts or with extracts or culture medium from HR C-, as the negative control. After cultivation for 24 h, extent of fungal growth was measured by enumeration of *Candida* cells through classical CFU counts. As shown in Fig. 5a, the multiplication of *C. albicans* in the presence of extracts from scFvFc 2G8 expressing HR was significantly reduced, in a dose-dependent fashion, as compared to growth in the presence of control extracts from HR C-. The same effect was seen when the fungus was cultured with the growth medium of the same HR clones or with the purified, HR-produced antibody (Fig. 5b). In these experiments, *Candida* growth inhibitory activity by the purified, HR-derived scFvFc 2G8 (200 μg/mL) as well as by the culture medium of the antibody expressing HR (concentration of scFvFc 2G8 150 μg/mL, as evaluated by ELISA) was fully comparable to that exerted by a reference preparation of the antibody, purified from *N. benthamiana* leaves and dissolved at a concentration of 200 μg/mL in HR C- culture supernatants (nearly 50% reduction of fungal growth).

## Discussion

In this work, we evaluated HR for the production of a chimeric scFvFc (scFvFc 2G8) directed against β1,3-glucan, a cell wall polysaccharide crucial for growth and survival of several fungal pathogens. The scFvFc molecule presents the advantage of being coded by a single sequence, making the plant transformation easier, while still able to dimerize (thanks to the hinge portion), retaining the binding avidity typical of an IgG. Accordingly, the scFvFc 2G8 proved to be able to inhibit in vitro and in vivo the opportunistic fungus *C. albicans* with an efficacy similar to that of the full IgG [14]. HR represent an easy-to-obtain platform mainly used as source of phytochemicals but also for production of recombinant proteins that can be directly secreted into the culture medium. This system has been chosen as an alternative to whole plant transient expression approaches, such as the widely used agroinfiltration, which ensure high yields but require more laborious and costly downstream purification procedures. Purification is indeed mandatory in a cGMP compliant process for proteins intended to be used in immunotherapy and, agroinfiltrated plant tissue implies more stringent purification than HR in order to eliminate not only plant-derived but also bacterial contaminants, that are absent in axenic HR cultures.

In our investigation, two different *Solanaceae* species were transformed to generate HR expressing scFvFc 2G8: *N. benthamiana*, already used to express Abs 2G8 by agroinfiltration, and *S. lycopersicum*, investigated here for the first time. Both species were successfully and easily transformed, obtaining a comparable numbers of total positive HR clones (26 for *N. benthamiana* and 23 for *S. lycopersicum*). Of interest, however, the percentage of clones with a high antibody expression level was much higher in *S. lycopersicum* (43%) than in *N. benthamiana* (12%). Although the difference was not statistically significant, this result suggests an issue requiring further comparative investigations on the two species.

As demonstrated by Western analysis, the scFvFc has been expressed and correctly assembled in HR of both *Solanaceae* and just few degradation products were found. Moreover, antibody yields from the best clones, quantified by functional ELISA, were satisfactory, ranging from 18 to 25 μg/g FW for *N. benthamiana* HR and up to 68 μg/g FW for *S. lycopersicum* HR. This yield for *N. benthamiana* is in line with that previously reported for an IgG expressed in HR of this species (31.3 μg/g FW) [19], while for tomato is definitely higher and not comparable with others because, to our knowledge, this is the first report on HR generated for antibody production in this host.

The scFvFc 2G8 expressed and purified from both *N. benthamiana* and *S. lycopersicum* hairy roots was characterized and showed to retain the functionality of the cognate antibody fragment produced transiently in *N. benthamiana* leaves. The SDS-PAGE analysis of both HR-purified 2G8 revealed a slightly different pattern with respect to the leaves-purified antibody. In particular, the non reducing-condition Coomassie-stained gel showed a major band corresponding to the assembled dimeric antibody and additional bands corresponding to minor degradation products, of which two prevalent at about 90 and 55 kDa. Almost certainly both bands derived from a proteolytic cleavage in the hinge region connecting constant domains (CH2-CH3) to scFv. This is not surprising since it was demonstrated that antibodies expressed in plants are particularly prone to proteolysis in the hinge region [20] and in a previous work, in which a scFvFc was expressed in HR of *N. benthamiana*, the purified antibody showed a similar degradation pattern [21].

HR offers the possibility to secrete and accumulate a recombinant protein in culture medium (rhizosecretion), yielding the desired product in a mixture with a sensibly lower content of unwanted plant-derived proteins, as compared to an extract from leaf tissues. However, in the root culture standard medium (a mixture of salts, micro and macronutrients) the scFvFc 2G8 was detectable at very low levels (*N. benthamiana*) or was undetectable at all (*S. lycopersicum*). This result was almost expected, since relatively low yields of rhizosecretion of recombinant proteins in the applied conditions have been already documented for other proteins [22]. Various strategies have been devised to improve rhizosecretion, acting either at genetic level [23, 24] or adding growth regulators [13, 21, 25], KNO_3_ as a source of nitrate to promote root growth, and auxins, that promote lateral and adventitious roots formation and increase roots/medium contact surface area [19, 26]. Stabilizers/protectors for secreted proteins, such as PVP, have also been added in some formulations [27, 28].

We thus adopted a culture medium enriched in plant growth regulators recently developed by Hakkinen and colleagues (2014), to optimize the secretion of an antibody in *N. tabacum* HR. Unfortunately, this medium was detrimental for the growth of both our *N. benthamiana* and, particularly, *S. lycopersicum* HR, due, probably, to the presence of PVP and of KNO_3_ and NAA, as auxin, at high concentrations. Since the main aim of our work is to use the extract or the growth medium of HR to obtain a preparation against pathogenic fungi, we decided to develop a different medium more suitable for our purposes, with minimal amounts of growth regulators but an enhanced antibody production. Indeed, removal of PVP and reduction of NAA and KNO_3_, allowed to trigger antibody secretion by *N. benthamiana* HR at concentrations up to ~ 7 mg/L, with a peak at 9 days post-induction. This yield was in the same order of magnitude of those already reported [19, 21, 22], in which, however, much more complex and rich media were used. The yields obtained in this work are even more satisfactory, considering the fact that quantification was made by a functional ELISA, detecting exclusively the presence of antigen-binding, intact antibody. Differently, in the only other report on a scFvFc antibody rhizosecretion [21], secreted antibody was quantified by an anti-γ chain antibody, detecting both unassembled Fc chains and degradation fragments, possibly leading to yield overestimation. Moreover, to increase the rhizosecretion of scFvFc, in our work, we have used a simplified approach, therefore the antibody yield could be further implemented, in the future, by a full factorial design approach [29, 30].

Furthermore, SDS PAGE analysis of the root secretome in our optimized medium showed that, although the total amount of secreted proteins was greater than that of the standard MS medium, it did not increase over time up to 9 days post-induction. The lower number of contaminating proteins represents a further advantage both for the direct use of the culture medium and for the possible subsequent antibody purification procedure.

As to *S. lycopersicum,* the few investigations on HR address issues related to the production of small molecules such as anthocyanins [31], vaccine antigens [32], or the evaluation of specific gene promoters [33] but in no case were used for antibody expression. Rhizosecretion in tomato HR was evaluated in a study reporting very low amounts of the protein of interest (LTB antigen), compared with other *Solanaceae* species (tobacco and petunia) investigated [12]. In the present work, we tried for the first time to obtain antibody rhizosecretion from tomato HR, without success, despite the fact that HR were easily generated and the antibody is well expressed inside root cells.

Since one of the aims of this work was to eliminate as much as possible purification steps, optimization of rhizosecretion and evaluation of antibody stability in the culture medium were strictly needful. In our system, we found that the HR culture medium per se affects protein stability, probably causing antibody precipitation. In fact, incubation of a purified scFvFc 2G8 either in our MS-basal medium (pH 5.8) or in buffered saline solution (pH 7.2) caused a substantial reduction in the amount of soluble antibody only for medium-incubated samples. This effect could be due either to the slightly acidic pH (5.8) of the medium, necessary for both complete solubilization of its components and for efficient nutrient assimilation by roots, or to some MS components, or to both factors, as suggested in a previous report [34], describing the aggregative effect on antibodies by low pH and high salt concentration. We also considered degradation issues due to presence of proteases into the culture medium. In fact, targeting antibody in the extracellular space offers advantages in terms of purification but, at the same time, exposes the protein to the proteolytic activity of plant cells peptidases. A genomic analysis of *Arabidopsis*, rice and *N. benthamiana* [35] revealed that a large proportion of plant peptidases are predicted to be targeted in extracellular space and the presence of peptidases was found in the hydroponic medium of tobacco plants expressing a human monoclonal antibody [26]. To date, little is still known about plant peptidases [36], thus we investigated the proteolytic activity in hairy roots of *N. benthamiana* and *S. lycopersicum*. Our results show a greater activity, in the culture medium of *S. lycopersicum* as compared to *N. benthamiana* HR, considerably influencing the integrity of the secreted antibody. The occurrence of such degradation reflects, albeit to a greater extent, what has already been observed in antibodies purified from root tissues. Moreover, a preliminary evaluation of which proteases were present in the growth medium was effected by gel zymography, demonstrating that the pattern of extracellular metalloproteinases appeared to be different in the two HR species. Considering that, besides Met peptidases, serine and cysteine are the most abundant class of secreted proteases in plants [37, 38], it is reasonable to assume that a greater number of proteases are differentially expressed, and supposedly involved in antibody degradation, in the two species. Our results confirmed that the proteolytic activity can vary amply in different plant species, as recently reported by Lallemand and colleagues [18].

The ability to inhibit per se, in the absence of host immune cells, the growth and multiplication of different fungal pathogens, featuring a sort of antibiotic action, is a distinctive biological activity of anti-β-glucan antibodies that crucially contributes to their antifungal protective action in vivo [14, 39, 40]. We show here that the scFvFc 2G8, purified from HR or present in concentrated total extracts and growth medium of *N. benthamiana* HR is fully functional and sufficient to exert a significant growth inhibitory effect in vitro on the fungal pathogen *C. albicans.* In particular, the antibody HR-produced showed a similar (or even slightly superior) potency if compared to that transiently expressed and purified from *N. benthamiana* leaves. These results clearly indicate that the anti-*Candida* activity of HR-secreted, “native” scFvFc 2G8 is fully preserved even in the presence of the rather complex mixture of other molecules, either plant-derived or not, present in HR protein extracts or culture medium.

## Conclusions

Two different *Solanaceae* species have been exploited successfully for production of the antifungal scFvFc 2G8. Hairy roots derived from these plants have been chosen as a synthesis source instead of leaves or full plants, offering additional advantages of containment and control over environmental factors.

The study and the characterization of these HR allowed us to optimize the antibody expression but also improve knowledge on the factors negatively affecting this system. Antibody instability in HR culture medium and quantitative and qualitative differences in extracellular proteolytic activity between the two HR species may explain, at least partially, the low yield of scFvFc 2G8 detected in *N. benthamiana* HR culture medium, as compared to levels recovered from the root cells, and the reason why no antibody could be found in tomato HR growth medium.

More important, we successfully utilized a root extract or HR culture medium to inhibit *C. albicans* growth, in view to reduce expensive purification steps currently required for a drug by strict regulations in the field of human medicine. Using HR platform, this process can be simplified with a considerable reduction in costs and time and, although further improvements are necessary, our work represent a step forward in recombinant antibody-based formulation for topical therapies.

## Methods

### Vectors and hairy root generation

The binary vector containing the scFvFc 2G8 (pBIM2G8) gene was obtained as described in a previous work [14]. A vector to be used for irrelevant control (pBIc-) was obtained by deleting the scFvFc-encoding sequence from the pBIM2G8 vector through SmaI/SacI digestion, followed by fill-in reaction mediated by Klenow enzyme and final ligation. For plant transformation, *R. rhizogenes* A4 (ATCC®15834™) cells, transformed with pBIM2G8 or pBIc- by electroporation, were grown, at 28 °C, 250 rpm, in YEB medium (yeast extract 1 g/L; beef extract 5 g/L; peptone 5 g/L; sucrose 5 g/L; MgSO_4_7H_2_0 2 mM; pH 7.2), up to an optical density of 1.0, then pelleted at 5,500 g for 10′ and resuspended in liquid MS medium (M0222, Duchefa) supplemented with 1 mM 3′,5′-Dimethoxy-4′-hydroxyacetophenone (acetosyringone). Leaf discs of *N. benthamiana* L*.* and *S. lycopersicum* L. (cv. Micro-Tom) plants, grown in a containment greenhouse and sterilized in 0.1% sodium hypochlorite, were incubated with the suspension of transformed *R. rhizogenes* for 15′. Transformed discs were then dried on sterile filter paper, placed upside down on petri dishes containing MS medium supplemented with 0.8% agar, 3% sucrose and 0.1 mM acetosyringone and finally incubated in the dark at 25 °C. After 2 days, explants were transferred on MS, 0.8% agar, 3% sucrose, supplemented with the antibiotics Cefotaxime (250 mg/L) to eliminate residual bacterial cells, and Kanamycin (100 mg/L) to select transformed plant cells. After 15 days, single roots were isolated from the explants and transferred in new individual petri dishes on the selective medium mentioned above for at least a month. *R. rhizogenes* was eradicated by transferring roots every 15 days onto MS agar plates using decreasing Cef concentrations (0.25, 0.125, and 0.05 g/L) until no antibiotic was added. The cultures were maintained in the dark, at 23 °C with a monthly subcultivation frequency. All the HR clones generated were screened for scFvFc 2G8 expression in the root tissue by a functional ELISA on beta-glucan, as described below. For each primary HR clone from both *N. benthamiana* and *S. lycopersicum*, the quantification was performed on roots grown for 14 days in MS 3% sucrose liquid medium, blotted on paper and frozen in liquid nitrogen before storing at − 80 °C. The procedure was repeated at least 3 times on roots sub-cultured up to 8 months after the transformation, to verify the antibody expression stability.

### Extraction of total soluble protein

Total soluble protein extracts were obtained from at least 200 mg of root from each HR clone. Roots were ground and homogenized in two volumes of ice-cold phosphate-buffered saline (PBS) pH 7.2 containing 1 mM phenylmethanesulfonyl fluoride (PMSF, Sigma-Aldrich) and 5 mM ethylenediaminetetraacetic acid (EDTA) pH 8. The extracts were clarified by centrifugation (10,000 g) for 10′ at 4 °C, the resulting supernatants were recovered and total protein concentration was evaluated through a Bradford assay [41].

### Quantification of scFvFc 2G8 by ELISA

Immuno MaxiSorp plates (NUNC) were coated with 100 μl of the β-glucan laminarin (Sigma-Aldrich) at a concentration of 100 μg/mL in 50 mM carbonate/bicarbonate buffer (pH 9.6) overnight at 4 °C. Plates were blocked with 2% BSA (w/v) in PBS (Fraction V, Sigma-Aldrich) for 2 h at 37 °C, washed with PBS containing 0.1% v/v TWEEN® 20 (Sigma-Aldrich) (PBST), and incubated for 1 h at 37 °C with the HR extracts or culture medium. For the initial screening of HR clones 10 μL of root extracts (corresponding to about 5 mg of root biomass) were analysed, while for quantification of scFvFc yield of the best clones 2 or 4 μL of extracts (corresponding to 1 and 2 mg of root biomass) were used. Different volumes of culture medium (generally 50–25 μL) normalized considering the root biomasses were analysed. When necessary, samples were diluted in PBS supplemented with 0.15% BSA (w/v) and 0.05% TWEEN® 20 (v/v). After washing with PBST, an alkaline phosphatase-conjugated goat anti-human IgG (γ-chain specific) antibody (A9544, Sigma-Aldrich), diluted 1:10,000 in sample buffer, was added to the wells and incubated for 1 h at 37 °C. Enzymatic activity was measured using the p-NitroPhenyl Phosphate substrate (Sigma-Aldrich) and read at 405 nm on a microtiter plate reader (Sunrise, TECAN) after 30 min of reaction. Each sample was assayed in triplicate and antibody concentration was calculated by interpolation of values obtained from a *N. benthamiana* leaf-purified scFvFc 2G8 [14] calibration curve. Extracts and culture medium from *N. benthamiana* and *S. lycopersicum* HRs transformed with *R. rhizogenes* harbouring the empty vector pBIc- were used as negative controls.

### Western blot

HR extracts normalized for TSP content (10 μg) were resolved on 10% SDS-PAGE acrylamide gels and blotted on a PVDF membrane (Millipore) using a Semi-Dry Transfer Unit (Hoefer TE70; GE Healthcare). After washing, membranes were blocked 2 h at RT in PBS with 5% (w/v) skimmed milk. ScFvFc 2G8 bands were detected by incubation with a horseradish peroxidase (HRP)-conjugated goat anti-human IgG (γ-chain specific) antibody (A8419, Sigma-Aldrich) diluted 1:5000 in PBS with 2% (w/v) skimmed milk, followed by extensive washings (once with PBS 0.1% TWEEN® 20 and twice in PBS) and development with the Immobilon Western chemiluminescence HRP substrate (Millipore). The ImageQuant™ LAS 500 (GE Healthcare) was used for chemiluminescence signal detection.

### Antibody purification from *N. benthamiana* and *S. lycopersicum* HR

Roots grown in MS, 3% sucrose, liquid culture for 3 weeks were ground and homogenized in two volumes of ice-cold phosphate-buffered saline (PBS) pH 7.2 containing 1 mM PMSF and 5 mM EDTA. The extract was centrifuged at 10,000 g, at 4 °C, for 10 min, the supernatant was filtered using Miracloth™ (Millipore) and centrifuged again at 30,000 g, at 4 °C, for 30 min. The scFvFc was purified from clarified extract, filtered through 0.45 μm syringe filters (Millipore), by protein A based affinity chromatography, which was carried out as previously described [14]. Briefly, the clarified culture medium was loaded onto a protein-A affinity column (1 mL HiTrap rProtein A FF; GE Healthcare) previously equilibrated with extraction buffer (PBS 1X) at a flow rate of 1 mL/min. The column was washed with 10 mL of PBS (10 column volumes), and the antibody was eluted with 0.1 M of citric acid, pH 3.2, and buffered with 1/5 volume of 1 M Tris-HCl, pH 8.0. Antibody concentration in the eluted fractions was determined spectrophotometrically by measuring the corresponding absorbance at 280 nm and antibody containing fractions were pooled and concentrated/dialysed against PBS using Amicon® Ultra-4, 50 kDa cutoff, (Millipore) following the manufacturer’s instructions. Purified antibodies were analysed by SDS-PAGE, followed by Coomassie staining.

### HR liquid culture and optimization of antibody rhizosecretion

To evaluate the growth kinetic in liquid culture of best expressing clones of *N. benthamiana* (clone #7) and *S. lycopersicum* (clone #11) HR, ~ 200 mg of roots where seeded in 250 mL Erlenmeyer flasks containing 10 mL of MS medium supplemented with 3% sucrose, kept in slight agitation (90 rpm) in the dark, at 22 °C, after 15 days, volume was increased to 30 mL with the same buffer. To optimize antibody rhizosecretion, 200 mg of HR where seeded in 150 mL Erlenmeyer flasks containing 4 mL of MS medium supplemented with 3% sucrose, in the same conditions as above. After 2 weeks of cultivation scFvFc 2G8 secretion in culture medium was triggered using, instead of basic MS, 4 mL of MS supplemented with 3% sucrose, 14 g/L of KNO_3_, 19 mg/L of NAA and 1.5 g/L of PVP (MW 360,000), as previously described for tobacco HR [13]. To optimize antibody rhizosecretion, different NAA and KNO_3_ concentrations (NAA, from 9.00 mg/L to 0.33 mg/L; KNO_3,_ 5 g/L and 10 g/L) were also experimentally tested, keeping unmodified the other supplements. After 4 days, the induction medium was totally replaced and HR were cultured for additional 5 days and an aliquot of each HR culture medium (200 μL) was collected from the spent medium at different time points (day 4, 7 and 9), after the addition of fresh MS with hormones. Yield and kinetics of Ab secretion, in the different experimental conditions (each of these in duplicate), were evaluated by quantifying scFvFc 2G8 by an indirect quantitative ELISA on immobilized β-glucan, as described above. These data were obtained from three independent HR cultures.

Proteins rhizosecreted by *N. benthamiana* HR C- in MS without growth regulators, in our optimized MS supplemented with KNO_3_ 10 g/L and NAA 3 mg/L and in the MS medium supplemented with KNO_3_ 12 g/L, NAA 19 mg/L and PVP 1.5%, sampled at 4, 7 and 9 days from growth regulators addition were analysed on 10% SDS-PAGE acrylamide gel followed by silver staining. Variable volume, normalized considering the biomass of HR liquid culture (corresponding to 25 mg of roots), were loaded on the gel.

### ScFvFc in vitro degradation and/or precipitation

*N. benthamiana* leaf-purified scFvFc 2G8 (1.5 μg) [14] was incubated at RT in 150 μL of MS medium (pH 5.8) or, as control, in PBS (pH 7.2) for 0, 4, 24 and 120 h. In parallel, the same amount of purified antibody was incubated for 4 and 24 h at RT in culture media of *N. benthamiana* or *S. lycopersicum* HRs transformed with A4 *R. rhizogenes* harbouring the pBIc- empty vector. The culture medium was collected from flasks containing a comparable quantity of root biomass after 3 weeks of HR growth. Samples of HR growth medium supplemented with protease inhibitors (1 mM PMSF and 20 mM EDTA) were used as a specificity control. At different time points, 15 μL of the antibody-HR medium mixtures (containing about 150 ng of scFvFc 2G8) were sampled and stored at − 20 °C. All samples were loaded on a reducing 10% SDS-PAGE acrylamide gel and the resolved proteins were visualized by Western blotting, as described above, to evaluate scFvFc integrity.

### Zymography

Gel zymography and rhizosecretome preparations from *N. benthamiana* and *S. lycopersicum* HR were performed as previously described [18]. Eighteen μL of HR culture medium concentrated 4 times using Amicon® Ultra-4, 10 kDa cutoff (Millipore) following the manufacturer’s instructions, or rhizosecretome preparations were analysed.

### Preparation of concentrated extracts and growth medium of *N. benthamiana* HR

Concentrated HR extract and growth medium were prepared from a culture of HR grown for about 4 weeks, following the developed protocol for antibody rhizosecretion, and sampled at 9 days after adding the optimized medium. The extracts were obtained as described above starting from 10 g of roots. The extract and culture medium were concentrated 15 and 25 times, respectively, using Amicon® Ultra-4, 10 kDa cutoff (Millipore), following the manufacturer’s instructions, and passed through a 0.22-μm filter. The amount of functional scFvFc 2G8 in both concentrated extract and culture medium was evaluated by ELISA.

### *Candida albicans* growth inhibition assay

These assays were performed essentially as reported previously [39]. Briefly, *C. albicans* cells, strain 3153 from the culture collection of Istituto Superiore di Sanità, Rome, Italy, were grown in the presence of concentrated extracts or culture medium of HR or purified scFvFc 2G8. Each condition was tested in triplicate cultures. Final *Candida* growth was evaluated by enumeration of fungal colony forming units (CFU), following a standard protocol. To this end, each single culture was serially diluted (10^2^, 10^3^, 10^4^, 10^5^ times) and triplicate samples of each dilution (0.1 mL) were spread onto solid Sabouraud agar medium in culture Petri dishes. After incubation for 24 h at 37 °C, number of fungal colonies grown on solid medium (corresponding to number of fungal cells present in the inoculated samples) were counted and number of fungal cells present in the experimental culture of origin were calculated taking into account the applied dilutions. Per cent growth inhibition values were calculated by comparing numbers of *Candida* CFU present in scFvFc 2G8-containing cultures with those of parallel negative control cultures. The fungal growth-inhibitory activity of the scFvFc 2G8-containing samples was always confirmed by microscopic examination.

### Statistical analysis

Statistical analysis was performed using the GraphPad Prism4 software. *P* values < 0.05 were considered statistically significant.

## Data Availability

The data and the materials produced are available from the corresponding author after reasonable request and signing a material transfer agreement.

## Abbreviations

CFU: Colony forming units
ELISA: Enzyme-linked immunosorbent assay
FW: Fresh weight
HR: Hairy root
IgG: Immunoglobulin G
MS: Murashige and Skoog medium
NAA: 1-naphtaleneacetic acid
PBS: Phosphate-buffered saline
PVP: Polyvinylpyrrolidone
scFvFc: Single-chain Fragment variable Fragment crystallisable
SDS-PAGE: Sodium dodecyl sulfate – polyacrylamide gel electrophoresis
